# Functional Polyimide/Polyhedral Oligomeric Silsesquioxane Nanocomposites

**DOI:** 10.3390/polym11010026

**Published:** 2018-12-25

**Authors:** Mohamed Gamal Mohamed, Shiao Wei Kuo

**Affiliations:** 1 Department of Materials and Optoelectronic Science, Center of Crystal Research, National Sun Yat-Sen University, Kaohsiung 80424, Taiwan; mgamal.eldin12@yahoo.com; 2 Chemistry Department, Faculty of Science, Assiut University, Assiut 71516, Egypt; 3 Department of Medicinal and Applied Chemistry, Kaohsiung Medical University, Kaohsiung 807, Taiwan

**Keywords:** polyhedral oligomeric silsesquioxane (POSS), double-decker-shaped silsesquioxane (DDSQ), polyimide, thermal stability, dielectric constant

## Abstract

The preparation of hybrid nanocomposite materials derived from polyhedral oligomeric silsesquioxane (POSS) nanoparticles and polyimide (PI) has recently attracted much attention from both academia and industry, because such materials can display low water absorption, high thermal stability, good mechanical characteristics, low dielectric constant, flame retardance, chemical resistance, thermo-redox stability, surface hydrophobicity, and excellent electrical properties. Herein, we discussed the various methods that have been used to insert POSS nanoparticles into PI matrices, through covalent chemical bonding and physical blending, as well as the influence of the POSS units on the physical properties of the PIs.

## 1. Introduction

**Figure 1:**
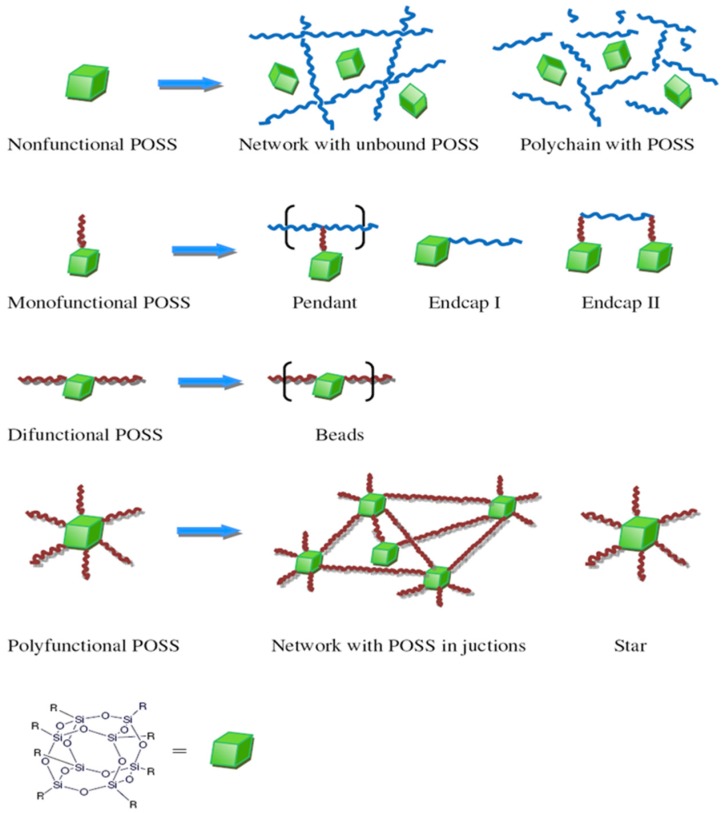
Schematic representation of possible architectures for incorporation of polyhedral oligomeric silsesquioxanes (POSS) nanoparticles (NPs) into polymer matrices [2]. Reproduced with permission from Elsevier.

Polyhedral oligomeric silsesquioxanes (POSS) form a class of nanostructured materials having diameters on the order of 1–3 nm; they can be considered as the smallest silica nanoparticles (NPs) [1,2,3,4]. Polyhedral oligomeric silsesquioxanes (POSS) moieties have the empirical formula (RSiO_1.5_)_n_, where R may be an organic functional group (e.g., alkyl, alkylene, epoxide unit, acrylate, hydroxyl) or a hydrogen atom, controlled porosity and nanometer sized structure; the structures of POSS NPs can be divided into partial cage, ladder, and random structures [5,6,7,8,9]. The properties of POSS-containing polymers can improve (e.g., decreased flammability, viscosity, and heat discharge, and increased rigidity, strength, and modulus) via the degree of dispersion of the POSS NPs into the polymer matrix [10,11,12,13,14,15,16,17,18,19,20,21,22,23,24,25,26]. Two general approaches have been used to incorporate POSS units into polymer matrices: (i) physical blending [27,28,29,30] and (ii) covalent attachment [29,30,31,32,33,34,35]. In the physical blending approach, the POSS NPs are blended (through melt-mixing or solvent-casting) with the polymers without forming covalent bonds; the success of this approach depends on the compatibility of the POSS NPs with the polymers [36,37,38]. In the covalent approach, the POSS NPs are attached to the polymer chain through covalent bonds. Although many types of POSS NP architectures (non-functional, mono-functional, bifunctional, and multi-functional) have been incorporated into polymer matrices (Figure 1), the preparation of polymer/POSS nanocomposites remains challenging because it can be expensive to perform on large scales, can require long equilibrium times, and can result in aggregation of the POSS NPs [39,40,41,42,43].

Polyimide (PI) is an important high-performance material because of its outstanding thermo-oxidative stability, chemical resistance, and mechanical and electrical stability [44,45,46]. Aromatic polyimides have been applied as insulating materials in many areas, including aerospace and microelectronics [47,48,49,50,51,52,53,54,55,56,57,58,59,60,61]. Aromatic PIs are polymers featuring stiff aromatic backbones; they are synthesized in two steps: condensation polymerization of an aliphatic or aromatic dianhydride acid and a diamine under ambient conditions in a dipolar aprotic solvent [e.g., *N*,*N*-dimethylacetamide (DMAc), *N*-methylpyrrolidinone (NMP), dimethylsulfoxide (DMSO)] to afford a corresponding poly(amic acid) (PAA), and subsequent ring closure [62,63,64,65]. Most aromatic PIs are insoluble and infusible because of their heteroaromatic structures and planar aromatic units. The thermal and mechanical properties of PIs can be enhanced, and their dielectric constants and linear coefficients of thermal expansion decreased, after the incorporation of various inorganic materials (e.g., silica NPs and ceramics) [65,66,67,68,69,70].

In this Review, we focused on the various methods that have been used to prepare PI/POSS hybrid nanocomposites based on Figure 1, including the blending of non-functional POSS NPs with PIs, the covalent linking of mono-functional POSS NPs with PIs at the chain end or side chain, the covalent incorporation of bifunctional POSS NPs into the PI main chain, and the formation of thermally crosslinked PIs with multi-functional POSS NPs. Additionally, we discussed the physical properties of the resulting PI/POSS nanocomposites, including their dielectric constants and dynamic mechanical, thermal, electrical, and surface properties.

## 2. PI/POSS Nanocomposites

### 2.1. Non-Functional POSS NPs Blended with PIs

**Figure 2:**
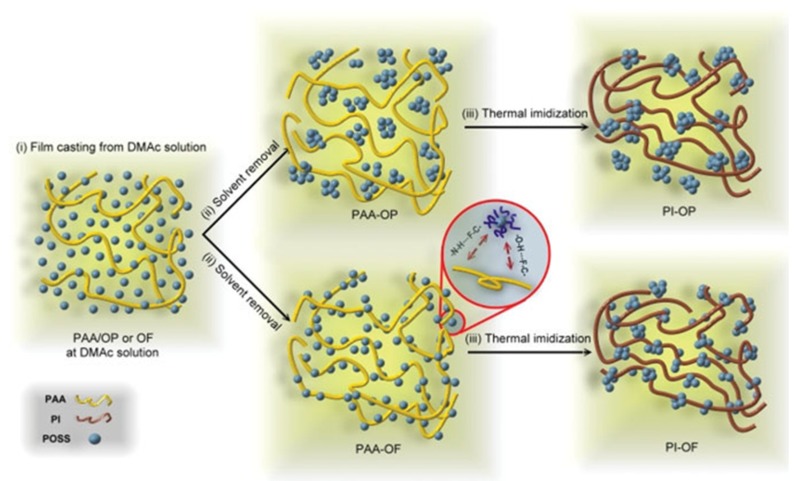
Cartoon representation of the deformation processes of Polyimide (PI)/octakis(dimethylsiloxyhexafluoropropyl) silsesquioxane (OF)-POSS and (OP)-POSS NPs during imidization [71]. Reproduced with permission from Wiley.

Chang et al. prepared PI/POSS nanocomposites exhibiting good thermal properties and low dielectric constants through the blending of fluorinated POSS precursors with PI [71]. The fluorinated POSS precursors was prepared by reacting octakis(dimethylsiloxyhexafluoropropyl) silsesquioxane (OF) with allyl 1,1,2,3,3,3-hexafluoropropyl ether (AHFPE) to form OF-POSS NPs, and with allyl propyl ether (APE) to form octakis(propyl ether) silsesquioxane (OP-POSS) NPs, using platinum 1,3-divinyl-1,1,3,3-tetramethyldisiloxane [Pt(dvs)] as the catalyst. Morphological studies revealed the possible occurrence of phase separation in these two PAA/OP and PAA/OF systems after a process of thermal imidization and solvent removal (Figure 2). Based on dynamic mechanical analysis (DMA), the glass transition temperature (*T*_g_) of the PI (371 °C) increased after introduction 10 wt % of the OF-POSS NPs. In contrast, after the incorporation of 15 wt % of the OP-POSS NPs, the values of *T*_g_ of the PI (359 °C) decreased relative to that of the corresponding pure PI (366 °C), due to the bulky and rigid POSS units coming into close contact with the polymer chain matrix and increasing the free volume around them [72,73].

**Figure 3:**
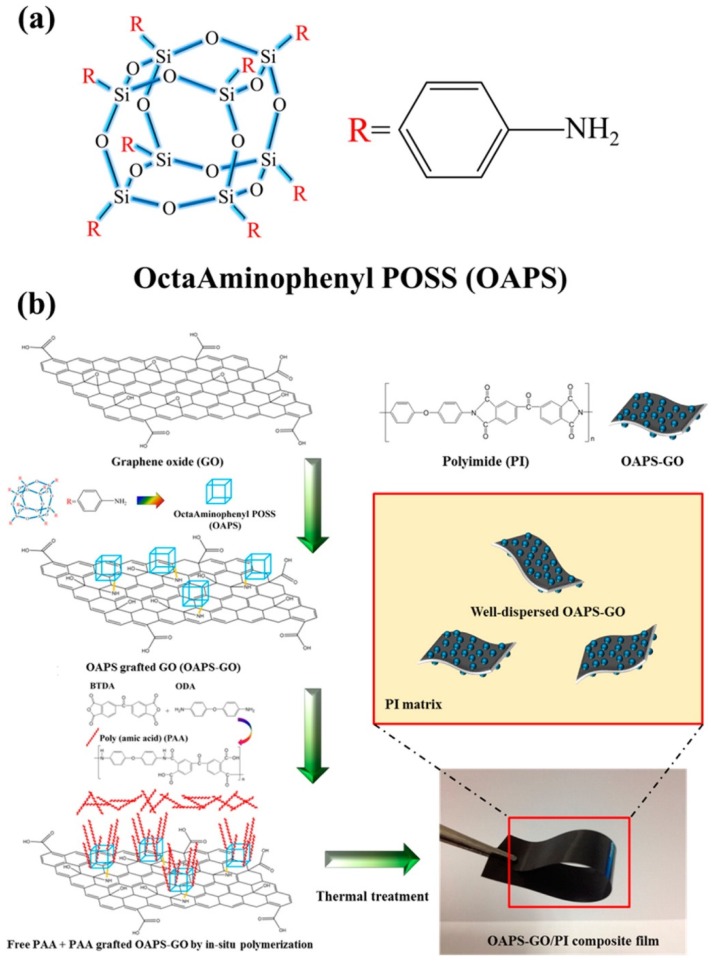
(**a**) Chemical structure of octa(aminophenyl)silsesquioxane (OAPS). (**b**) Schematic representation of the preparation of OAPS-graphene oxide (GO)/PI hybrid nanocomposites [74]. Reproduced with permission from the American Chemical Society.

**Figure 4:**
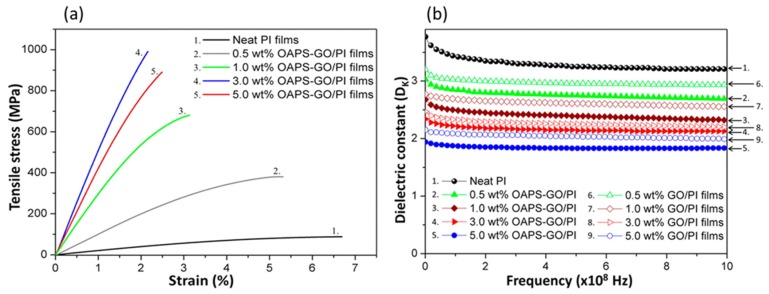
(**a**) Stress–strain curves of neat PI films and OAPS-GO/PI films with different contents of various amounts of OAPS-GO. (**b**) Profile dielectric constants of neat PI films, GO/PI films, and OAPS-GO/PI films [74]. Reproduced with permission from the American Chemical Society.

**Figure 5:**
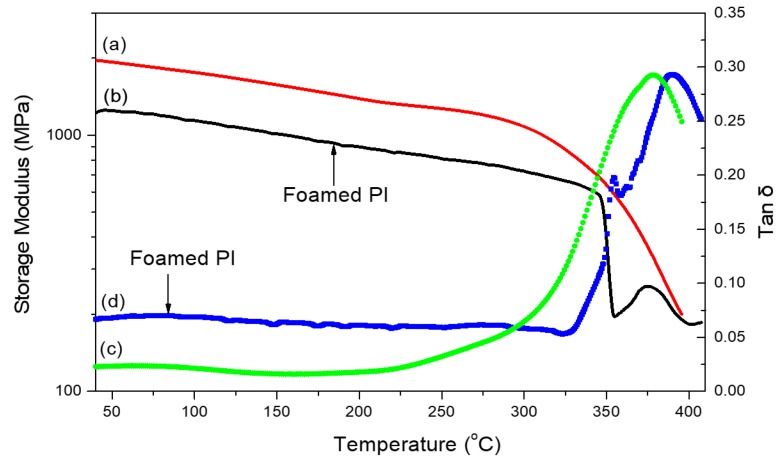
Dynamic mechanical analysis (DMA) curves for (**a**,**c**) pure PI and (**b**,**d**) porous PI (obtained from 10 wt % PEO-POSS), recorded at a heating rate of 2 °C/min [75]. Reproduced with permission from Elsevier.

Liao et al. [74] prepared octa(aminophenyl)silsesquioxane (OAPS)-functionalized graphene oxide (GO)–reinforced PI nanocomposite films (Figure 3). The tensile strength of the OAPS-GO/PI composite increased 11.2-fold after adding 3 wt % OAPS-GO (Figure 4(a)). In addition, the dielectric constant of the OAPS-GO/PI composite film decreased upon increasing the concentration of OAPS-GO (Figure 4b). Furthermore, we have prepared nanoporous PI films of low dielectric constant (*k* = 2.25) through blending PI with a star poly(ethylene oxide) PEO-POSS hybrid as a template and then removing the PEO segments through oxidative thermolysis to produce voids inside the polymer matrix (pore sizes: 10–40 nm) [75]. Figure 5 displays the storage moduli and values of tan δ for the pure PI and the porous PI templated by the star PEO-POSS NPs. Through DMA, the pure PI displayed a single value of *T*_g_ of 370 °C, while the porous PI exhibited two *T*_g_ at 360 and 385 °C. The lower value of *T*_g_ corresponded to that typically observed for micro-phase separation from the porous PI, suggesting that the storage modulus decreased as a result of its foam structure. These foam structures would have collapsed, such that the corresponding porous structures would no longer exist, at higher temperatures, with the residual silica from the star PEO-POSS after thermal calcination enhancing the value of *T*_g_ (385 °C) of the porous PI. More importantly, the dielectric constant decreased significantly, from 3.25 to 2.25, for this porous PI matrix.

### 2.2. Mono-Functional POSS NPs with PIs

#### 2.2.1. PIs Containing POSS NPs at Polymer Chain End

**Figure 6:**
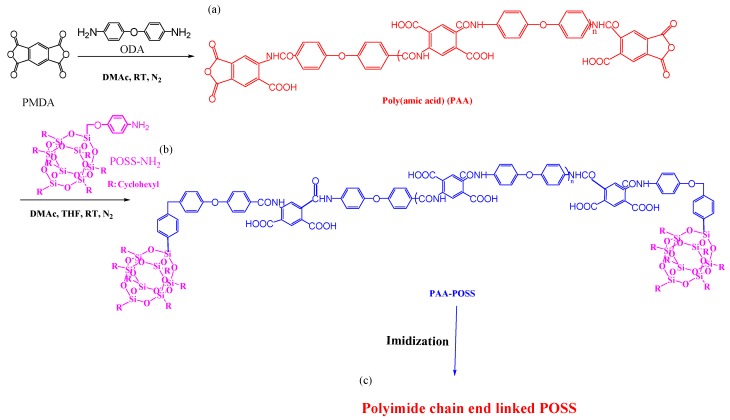
Preparation of (**a**) poly(amic acid) (PAA); (**b**) PAA/POSS nanocomposites; and (**c**) PI linked through its chain ends to POSS [76]. Reproduced with permission from the American Chemical Society.

Wei et al. [76] prepared nanoporous PI/POSS nanocomposites through a multistep process (Figure 6). First, pyromellitic dianhydride (PMDA) reacted with 4,4′-oxydianiline (ODA) in DMAc under a N_2_ atmosphere at room temperature to afford PAA. Then, POSS-NH_2_ reacted with PAA in DMAc to obtain PAA/POSS nanocomposites. The PI linked through its chain ends to POSS NPs was obtained after thermal imidization of the PAA/POSS nanocomposites at 300 °C. The dielectric constant of the resultant polyimide POSS nanocomposites decreased from 3.40 for the neat PI to 3.09 after adding 2.5 mol % of POSS units; this behavior was attributed to the phase-separated system and the uniformly porous structure (nanometer-scale) of the POSS molecules. In addition, the resultant PI linked through its chain ends to the POSS NPs formed lamellae or cylinders that were in the range of 60–70 nm long and 5 nm wide (through transmission electron microscope (TEM) image).

**Figure 7:**
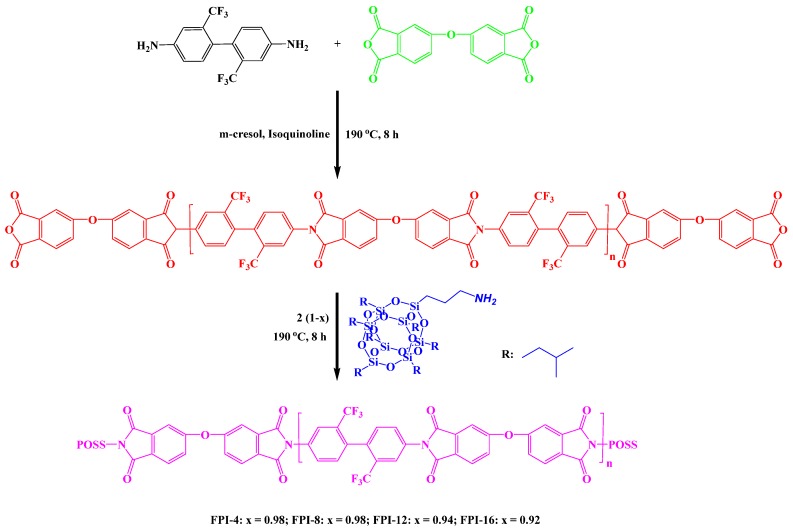
Synthesis of fluorinated PI/POSS hybrid nanocomposites [77]. Reproduced with permission from Springer.

Zhao et al. [77] synthesized a series of fluorinated PI/POSS hybrid materials through a simple route from 2,2′-bis(trifluoromethyl)benzidine, 4,4′-oxydiphthalic dianhydride, and a monofunctional POSS in m-cresol and isoquinoline as solvents (Figure 7). These hybrid polymers possessed low dielectric constants (in the range 2.47–2.92), high thermal stability, film formation ability, excellent solubility, and good hydrophobic and mechanical properties.

#### 2.2.2. PIs presenting POSS NPs Grafted to the Side Chains

**Figure 8:**
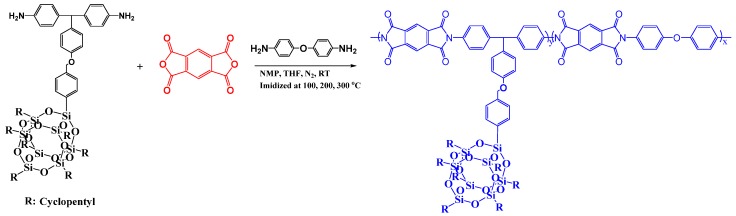
Schematic representation of side chain-tethered PI/POSS nanocomposites [78]. Reproduced with permission from the American Chemical Society.

Wei et al. [78] prepared PI-tethered POSS hybrid materials through copolymerization of a POSS-diamine, ODA, with PMDA (Figure 8). When the POSS content was 16 mol %, this PI/POSS composite displayed the large-scale self-assembled layer-by-layer structure of POSS. This layer-by-layer structure of POSS possessed (as elucidated by TEM observations) a layer length of greater than 100 nm and a layer spacing of 2–4 nm. Chen et al. [79] prepared hybrid film materials having tunable dielectric constants, lower than that of the neat PI, through thermally initiated free-radical graft polymerization of methacrylcyclopentyl-POSS (MA-POSS) with ozone-pretreated poly[*N*,*N*-(1,4-phenylene)-3,3′,4,4′-benzophenonetetracarboxylic amic acid] and subsequent thermal imidization to afford PI-*g*-PMA-POSS nanocomposite films. Nuclear magnetic resonance (NMR) spectroscopy, X-ray diffraction (XRD), and thermogravimetric analysis (TGA) confirmed the chemical composition and structure of these PI-*g*-PMA-POSS nanocomposite films.

### 2.3. Bifunctional POSS NPs Incorporated within the Main Chain of PIs

**Figure 9:**
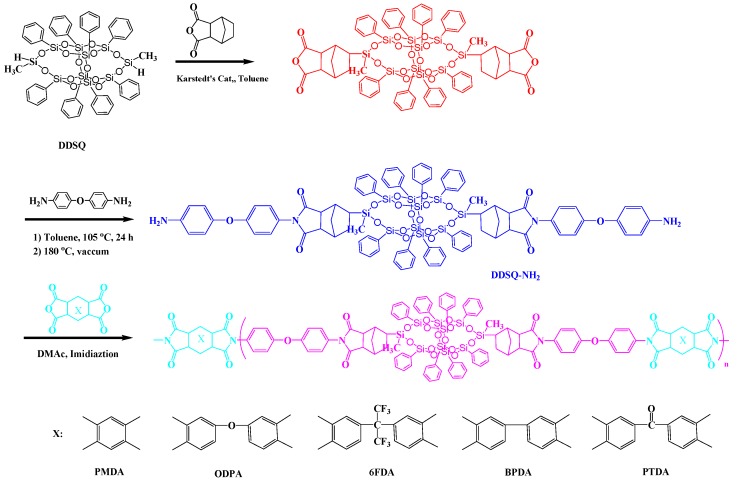
Preparation of semi-aromatic PIs containing a double-decker-shaped silsesquioxane (DDSQ) in the main chain [80]. Reproduced with permission from the American Chemical Society.

**Figure 10:**
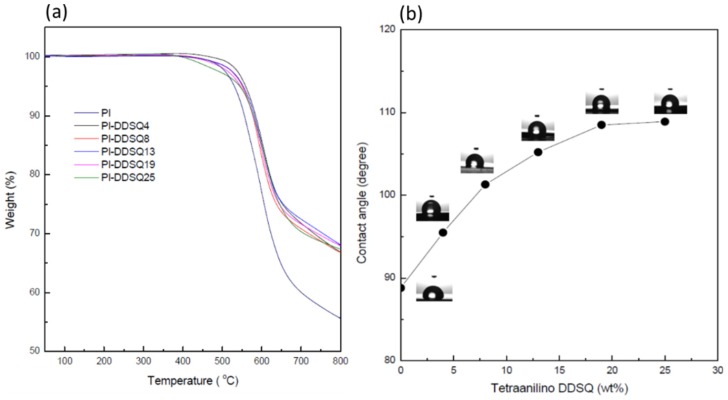
(**a**) Thermogravimetric analysis (TGA) thermogram and (**b**) water contact angles of PIs containing various contents of 5,11,14,17-tetranilino DDSQ [81]. Reproduced with permission from Wiley.

**Figure 11:**
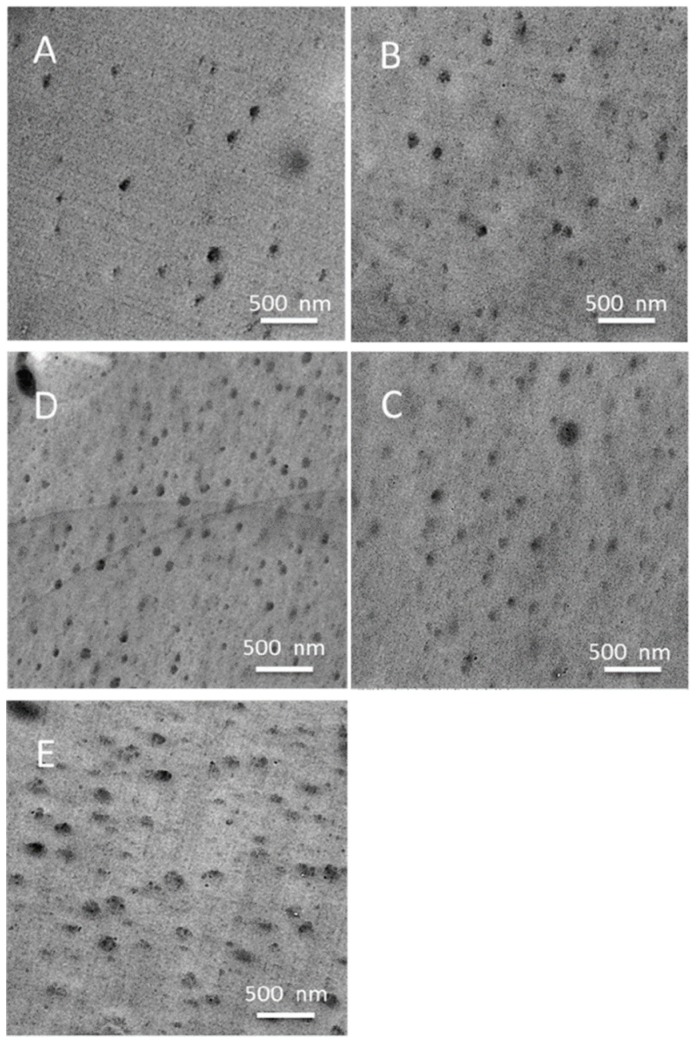
TEM micrographs of the synthesis of PIs containing various contents of 5,11,14,17 tetranilino DDSQ [81]. Reproduced with permission from Wiley.

Kakimoto et al. [80] synthesized various semi-aromatic PIs containing double-decker-shaped silsesquioxane (DDSQ) in the main chain (POSS-PIs) through the reactions of DDSQ-diamine, which was prepared through hydrosilylation of DDSQ with cis-5-norbornene-endo-2,3-dicarboxylic anhydride and a subsequent reaction with ODA. The DDSQ-NH_2_ reacted with various aromatic tetracarboxylic dianhydrides to obtain POSS-PIs nanocomposites (Figure 9). The POSS-PIs had low water absorption, low dielectric constants, and good thermal stabilities. In addition, the polymer hybrid materials films had good mechanical properties, with an elongation at breakage of 2.9–6.0%. Zheng et al. [81] synthesized a PI containing a tetrafunctional POSS through thermal imidization of 5,11,14,17-tetranilino DDSQ with 4,4′-diaminophenylether (ODA) and 3,3′,4,4′-benzo phenonetetracarboxylic dianhydride (DTDA) in DMAc, affording organic/inorganic PI nanocomposites incorporating variable contents of POSS units. Based on TGA and surface contact angle measurements (Figure 10a,b), these nanocomposite materials exhibited greater thermal stability and surface hydrophobicity, relative to those of the unmodified PI, upon increasing the POSS content. TEM imaging revealed (Figure 11) that the POSS molecules self-assembled into spherical microdomains having diameters in the range from 40 to 80 nm.

**Figure 12:**
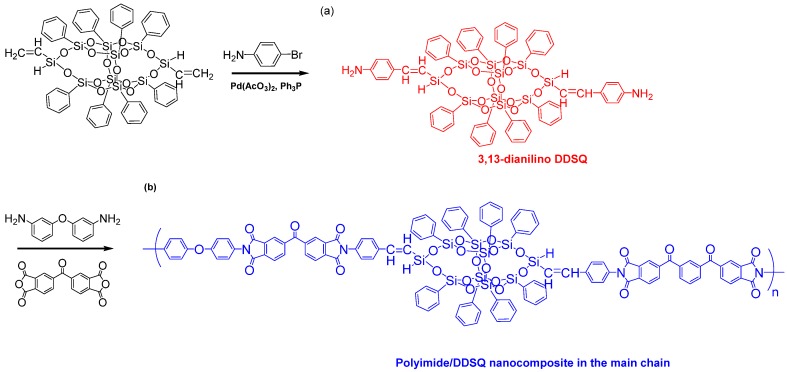
Preparation of organic/inorganic PIs with DDSQ in the main chain [82]. Reproduced with permission from the Royal Society of Chemistry.

Zheng et al. [82] reported a facile synthetic method for the preparation of organic/inorganic PI with DDSQ in the main chain (Figure 12). First, a DDSQ-diamine was synthesized through a Heck reaction of 3,13-divinyloctaphenyl double-decker silsesquioxane (3,13-divinyl DDSQ) with 4-bromoaniline in the presence of a palladium catalyst; next, thermal imidization of the 3,13-dianilino DDSQ with ODA and 3,3′,4,4′-benzophenonetetracarboxylic in DMAc afforded PI/DDSQ nanocomposites.

**Figure 13:**
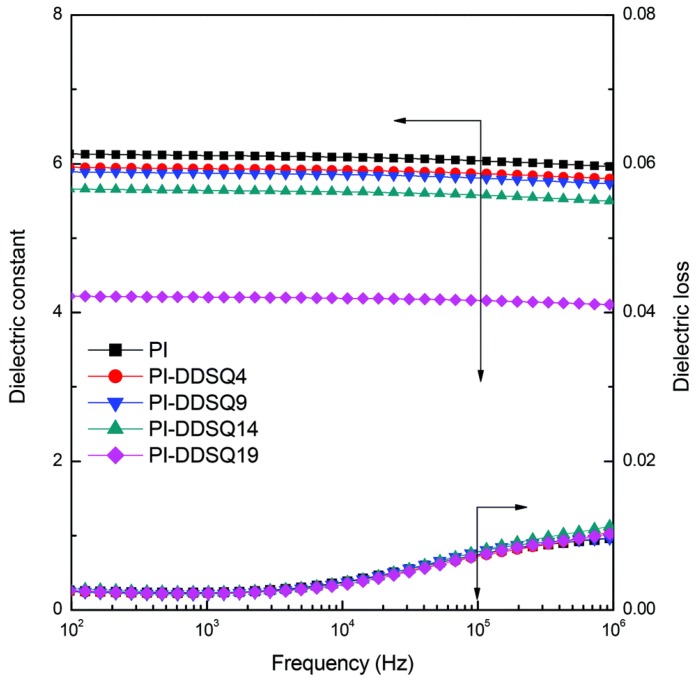
Plots of dielectric constant of organic/inorganic PIs with DDSQ in the main chain [82]. Reproduced with permission from the Royal Society of Chemistry.

According to the dielectric analysis, the dielectric constant of the PI/DDSQ nanocomposites decreased upon incorporating larger amounts of the DDSQ into the main chain (Figure 13). Furthermore, the thermal stability and surface hydrophobicity of the organic/inorganic nanocomposites were enhanced after the inclusion of the DDSQ in the main chain.

### 2.4. Thermally Crosslinked PIs Incorporating Multi-Functional POSS NPs

**Figure 14:**
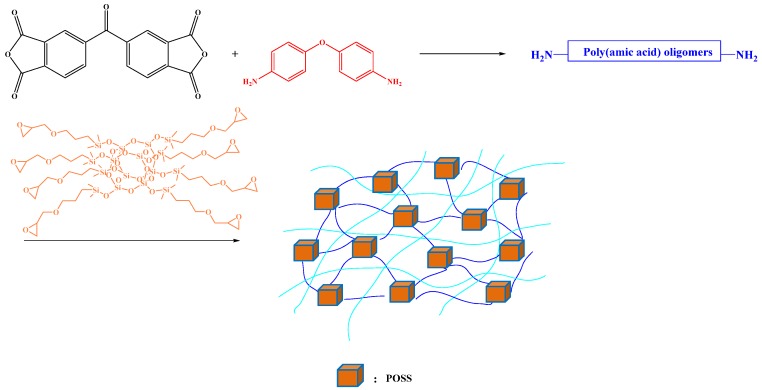
Preparation of OG-POSS cross-linked PIs [83]. Reproduced with permission from Elsevier.

We have prepared well-defined PI hybrid materials containing POSS NPs through the copolymerization of octakis(glycidyldimethylsiloxy)octasilsesquioxane (OG-POSS), 4,4′-carbonyldiphthalic anhydride (BTDA), and 4,4′-oxydianiline diamine (ODA) (Figure 14) [83]. The PI-POSS hybrid materials had low dielectric constants and thermal expansion coefficients that decreased from 66.23 to 63.28 to 58.25 ppm/°C when the POSS content increased from 0 to 10 wt %, presumably because of the increase in free volume caused by the presence of the POSS-tethered network. 

**Figure 15:**
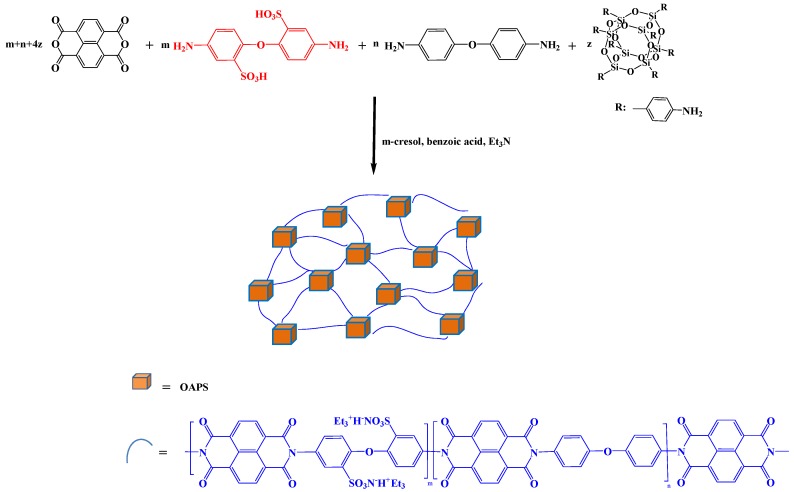
Preparation of POSS cross-linked cross-linked sulfonated PIs (SPIs) [84]. Reproduced with permission from Elsevier.

Li et al. prepared a series of octa(aminophenyl)silsesquioxane (OAPS) covalently cross-linked sulfonated PIs (SPIs) (Figure 15) [84]. These OAPS cross-linked PI membranes possessed greater chemical and thermal stability, higher strength, and excellent solution processability when compared with those of the linear sulfonated PI membrane. Moreover, when the OAPS content was 0.4 mol %, the proton conductivity of the cross-linked SPI membrane reached its highest value (0.111 S/cm).

**Figure 16:**
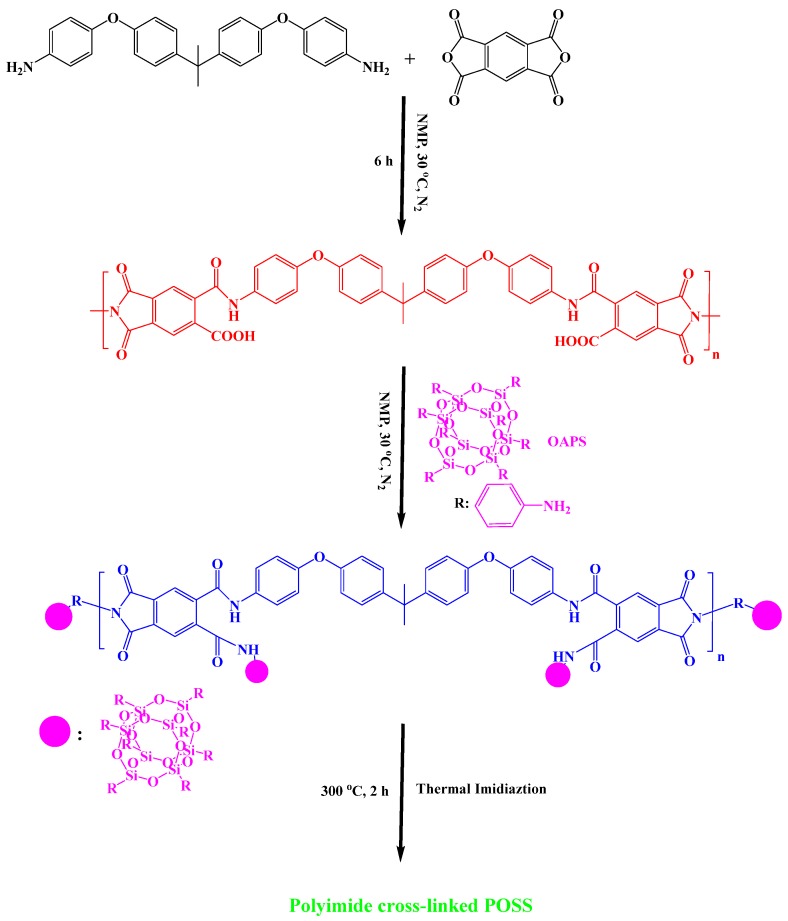
Schematic representation of PI cross-linked POSS [85]. Reproduced with permission from Elsevier.

**Figure 17:**
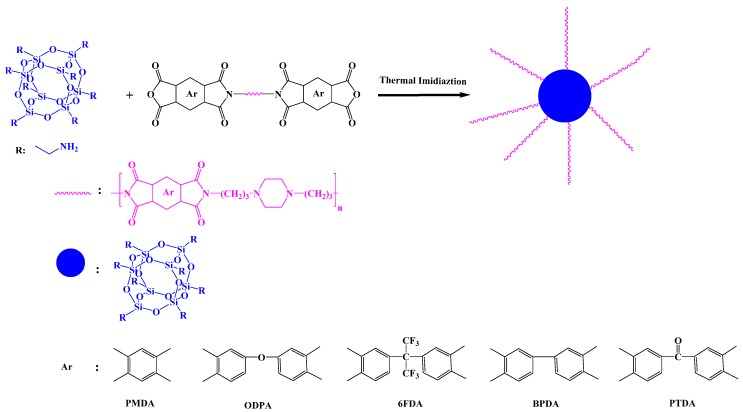
Preparation of star POSS/PI hybrid materials [86]. Reproduced with permission from Elsevier.

Alagar et al. synthesized PI/POSS hybrid) materials through a two-step method (Figure 16) [85]. First, the formation of PAA from bisphenol-A ether diamine (BAED) and PMDA in NMP under N_2_ at 30 °C, and its blending with various percentages of OAPS; subsequently, thermal imidization of the PAA/OAPS nanocomposites at 300 °C to obtain the POSS-PI nanocomposites. Thermal studies revealed that the glass transition temperature, char yield, thermal stability, and flame-retardant properties of the POSS-PI hybrid nanocomposite were all greater than those of the pristine PI. Adıguzel et al. [86] prepared star polyimides containing polyhedralsilsesquioxane as the core through in situ thermal curing of PAA with octa(aminopolyhedralsilsesquioxane) (Figure 17). The POSS-PI star nanocomposites exhibited unique characteristics including low water absorption, low dielectric constant, and good mechanical and thermal properties arising from the insertion of the rigid POSS units in the PI backbone.

## 3. Conclusions

In this Review, we discussed various preparation types of PIs containing POSS NPs that displayed attractive characteristics, including low dielectric constants, low water absorption, and methanol permeability; excellent mechanical and electrical properties; and high thermal stability, surface hydrophobicity, flame retardance, chemical resistance, proton conductivity, and thermo-redox stability compared with those of their pristine PI precursors. The insertion of these POSS units into the PI precursors has been performed through either physical blending or covalent bonding. The preparation of organic/inorganic hybrid materials containing POSS and PI remains an interesting topic in academic and industrial science because of their potential applications in fuel cells and gas separation membranes, as insulator materials in microelectronics, and in the aerospace industry.
